# The drug risks of cilostazol: A pharmacovigilance study of FDA Adverse Event Reporting System database

**DOI:** 10.1371/journal.pone.0314957

**Published:** 2024-12-04

**Authors:** Lufeng Peng, Xueli Li, Junhai Li, Shibin Liu, Gang Liang

**Affiliations:** 1 Qilu Hospital of Shandong University, Jinan, Shandong Province, China; 2 Dongying People’s Hospital (Dongying Hospital of Shandong Provincial Hospital Group), Dongying, Shandong Province, China; 3 Shandong University of Traditional Chinese Medicine, Jinan, Shandong Province, China; 4 Second Affiliated Hospital of Shandong University of Traditional Chinese Medicine, Jinan, Shandong Province, China; 5 Affiliated Hospital of Shandong University of Traditional Chinese Medicine, Jinan, Shandong Province, China; Al Nasiriyah Teaching Hospital, IRAQ

## Abstract

**Objective:**

Cilostazol is indicated for alleviating intermittent claudication (IC) in stable-phase peripheral arterial disease (PAD) patients. Conducting data mining on adverse events (AEs) of cilostazol in the U.S. Food and Drug Administration (FDA) Adverse Event Reporting System (FAERS) database to explore its potential medication risks and advance more rational and secure clinical medication practices.

**Methods:**

This study utilized the Open Vigil 2.1-MedDRA tool to retrieve and extract AE reporting data related to cilostazol from the FAERS database spanning the first quarter of 2004 to the first quarter of 2024. The primary methodology employed was the application of the reporting odds ratio (ROR) method to detect risk signals associated with AEs of cilostazol.

**Results:**

A total of 2,130 AE reports involving cilostazol were identified as the primary suspect drug, with a total of 7,134 AEs reported. These reports were predominantly concentrated among patients aged 60 and above, with a higher occurrence in males compared to females. Japan ranked first among the reporting countries, and the majority of reports were submitted by healthcare professionals. Through the screening of cilostazol, a total of 323 positive risk signals for AEs were identified, encompassing 23 system organ classes (SOCs). A comparison with the existing cilostazol product label revealed 8 AEs that were not included based on the number of AE reports, and 19 AEs that were not included based on the strength of the risk signals. Cilostazol exhibited positive risk signals for AEs primarily affecting 8 organ systems based on the SOC classification. Among these, cardiac disorders ranked highest, with a total of 53 positive risk signals for cardiovascular-related AEs identified. In terms of the number of reports, cardiac failure ranked first, aligning with the black box warning issued by the FDA regarding cilostazol. The occurrence of adverse reactions related to cilostazol is primarily concentrated within the first month of treatment. However, a certain proportion of adverse reactions have been reported to occur after long-term use (exceeding 360 days) of cilostazol therapy.

**Conclusion:**

Our results have further enriched the observations from existing clinical and real-world studies, uncovering new AE signals for cilostazol, including fall, cerebral infarction, pneumonia, loss of consciousness, acute kidney injury, renal impairment, renal failure, cardiac vein perforation, basal ganglia haematoma, cerebral hyperperfusion syndrome, et al. This study also highlights the significant impact of cilostazol on the cardiovascular system, necessitating close attention to potential cardiovascular toxicities. In addition to focusing on the short-term adverse reactions following cilostazol administration, thorough research into its long-term safety profile is also imperative. This study provides recommendations and guidance for the rational and safe clinical use of cilostazol. In the future, prospective studies are needed to explore the occurrence of related AEs further.

## 1 Introduction

Intermittent claudication (IC) refers to leg muscle pain, cramping, and fatigue caused by insufficient blood supply during walking or physical activity. The symptoms are relieved after rest and are the primary manifestation of peripheral arterial disease (PAD) [1]. PAD has a negative impact on quality of life. PAD is a disease characterized by atherosclerosis in the lower limb arteries, with IC being a crucial hallmark of atherosclerosis. It elevates the risk of cardiovascular diseases, with up to 60% of IC patients showing significant coronary and/or carotid artery lesions. Additionally, 40% of IC patients die or experience a stroke within five years of symptom onset [2,3]. The primary goal of intervention for IC is to alleviate symptoms and reduce systemic cardiovascular complications. While exercise therapy is one of the most effective conservative treatments for intermittent claudication, optimal achievement of treatment goals to improve quality of life and reduce amputation rates can be realized through pharmacological interventions that enhance walking capacity. In the development of IC treatment, active non-pharmacological interventions and pharmacotherapy targeting IC risk factors have been considered [4,5]. In the next two years, large-scale clinical trial results will become available for drugs targeting the stabilization and reversal of atherosclerosis, such as statins and angiotensin-converting enzyme inhibitors, antiplatelet drugs, recombinant growth factors, and immunomodulators, which can be used for IC treatment [6].

Cilostazol, a pharmacological agent, belongs to the class of selective inhibitors targeting phosphodiesterase 3 enzymes found in platelets and vascular smooth muscle cells. With its potent antiplatelet and vasodilatory properties, cilostazol exhibits the ability to regulate cyclic adenosine monophosphate (cAMP) levels within these cells. Consequently, it effectively mitigates vascular proliferation, while also demonstrating intrinsic lipid-lowering effects within the body [7]. Recent multicenter, randomized, placebo-controlled trials have led to the approval of cilostazol by the U.S. FDA for the relief of intermittent claudication in stable PAD patients [8,9]. In these studies, cilostazol has been shown to double the walking distance compared to placebo, improve quality of life, and enhance overall patient well-being. Cilostazol possesses properties that encompass antiplatelet, antithrombotic, and vasodilatory characteristics. In vitro, ex vivo, and in vivo studies have demonstrated that cilostazol effectively inhibits platelet aggregation induced by ADP, collagen, arachidonic acid, adrenaline, thrombin, lipoprotein remnants, and shear stress, while not affecting bleeding time [10,11]. In animal models, cilostazol exhibits the ability to inhibit thrombus formation induced by various stimuli. The vasodilatory effect of cilostazol is attributed to its inhibition of calcium-induced contraction in smooth muscle cells (SMCs), without exerting a direct influence on contractile proteins [12]. Following a 6-week treatment of twice-daily administration of 100 mg of cilostazol in patients with intermittent claudication, there was a significant improvement observed in leg skin temperature, ankle blood flow, and skin blood flow (P < 0.05) [7]. Cilostazol is generally well-tolerated in clinical trials. Summary data on tolerability from placebo-controlled clinical trials indicate that headache, diarrhea, abnormal bowel movements, pain, infection, pharyngitis, rhinitis, peripheral edema, and nausea are the most commonly reported AEs, occurring in 5% or more of cilostazol recipients (n = 1301). However, discontinuation of cilostazol treatment has been observed in patients due to diarrhea, palpitations, or myocardial infarction [13].

The U.S. Food and Drug Administration(FDA) Adverse Event Reporting System (FAERS) database, open to the public, is a spontaneous reporting system (SRS) that comprises a wide range of case reports documenting adverse events(AEs) associated with various drugs. The FAERS database is publicly accessible and includes adverse reaction reports voluntarily submitted by healthcare professionals, drug manufacturers, and patients themselves from many countries worldwide. The data in the database are updated quarterly [14]. FAERS serves as the largest global repository for pharmacovigilance and is a valuable resource for detecting AEs related to drug use [15]. However, the effectiveness and safety information regarding drugs other than cilostazol mainly rely on clinical trials and meta-analyses, and there is currently a lack of comprehensive safety data from large-scale and real-world cohorts. Due to its extensive clinical application and the necessity of AE assessment, this study utilizes the latest reported data from the FAERS database to conduct pharmacovigilance analysis on cilostazol, providing valuable insights for its safe and rational clinical use.

## 2 Methods

### 2.1 Study design and data sources

The data for this study were derived from the FAERS drug AE reports database, covering the period from the first quarter of 2004 to the first quarter of 2024. The inclusion criteria for this study were as follows: (1) Performing a fuzzy search using the brand name and generic name of cilostazol (Pletal, Cilostazol) to select AE reports in which cilostazol was identified as the primary suspect drug; (2) The FAERS data files contained seven types of datasets: patient demographic and administrative information (DEMO), drug information (DRUG), coded for the adverse events (REAC), patient outcomes (OUTC), report sources (RPSR), therapy start dates and end dates for reported drugs (THER), and indications for drug administration (INDI), and deleted cases. Although the majority of the submitted reports originated from the United States, reports from any country were included in the analysis.

Indeed, there may be inconsistencies in the naming of drugs and AEs in the database. The symptoms of AEs were encoded using the Medical Dictionary for Regulatory Activities (MedDRA: https://www.meddra.org/), which is an internationally standardized and clinically validated terminology [16]. MedDRA was used to standardize the system organ class (SOC) and preferred term (PT) for the collected AEs. In this study, the Open Vigil 2.1-MedDRA tool (http://h2876314.stratoserver.net:8080/OV21d2/search/) was employed to retrieve and extract relevant data [17]. Due to the quarterly updates of the database, inevitable repetitions of previous public reports occur, necessitating reprocessing. Following FDA recommendations, a duplicate data removal process should be conducted prior to statistical analysis. In cases where CASEID matches, select the most recent FDA_DT; when both CASEID and FDA_DT match, prioritize the higher PRIMARYID [18,19].

Additionally, in this study, we focused on the analysis of AEs related to the specific indication (intermittent claudication). We only included reports submitted by healthcare professionals, including physicians, pharmacists, and other healthcare providers, to ensure the reliability of the results.

### 2.2 Statistical analysis

Signal detection for AEs: Signal detection was performed using the method of disproportionality analysis (DPA), employing the reporting odds ratio (ROR) and the proportional reporting ratio (PRR) for signal detection [17,20]. This method is based on a four-fold table (Table 1) and aims to identify potential AE signals by comparing the proportion of target events associated with the target drug to the proportion of target events associated with all other drugs. The ROR method utilizes a two-sided test with a 95% confidence interval (CI), where a lower limit greater than 1 indicates a signal, provided that the number of reports (a) is equal to or greater than 3. For the PRR method, the signal generation criteria include a minimum number of reports (a) of 3, a PRR value of 2 or higher, and a variance (χ2) of 4 or higher. The selected signals need to meet the criteria of at least one of the two methods, indicating a potential association between the drug and the event (Table 2). Count data is described using case numbers and proportions. All statistical analyses and visualizations were performed using R software (https://www.r-project.org/; version 4.0.0).

**Table 1:** Four-fold table of disproportionality methods.

Item	Number of target adverse event reports	Number of other adverse event reports	Total
**Target drug**	*a*	*b*	*a+b*
**Other drugs**	*c*	*d*	*c+d*
**Total**	*a+c*	*b+d*	*N = a+b+c+d*

**Table 2:** Formulas and threshold values of ROR and PRR.

Methods	Formula	Threshold value
**ROR**	ROR = (a/c) / (b/d) = ad / bc 95% CI = e^lnROR ± 1.96[(1/a) + (1/b) + (1/c) + (1/d)]^	*a*≥3; A signal is generated if the lower limit of 95%Cl of ROR>1.
**PRR**	PRR = [a/(a+b)] / [c/ (c + d)] 95% CI = e^lnPRR ± 1.96 {(1/a)—[1/ (a+b)] + (1/c)—[1/(c+d)]}^ χ2 = [a-N(a)]2/N(a) + [b-N(b)]2/N(b) + [c-N(c)]2/N(c) + [d-N(d)]2/N(d)	*a*≥3; PRR≥2, χ^2^≥4, a signal is generated.

Note: ROR, reporting odds ratio; PRR, proportional reporting ratio.

## 3 Results

### 3.1 Descriptive analysis

#### 3.1.1 Search target AE reporting process

**Fig 1:**
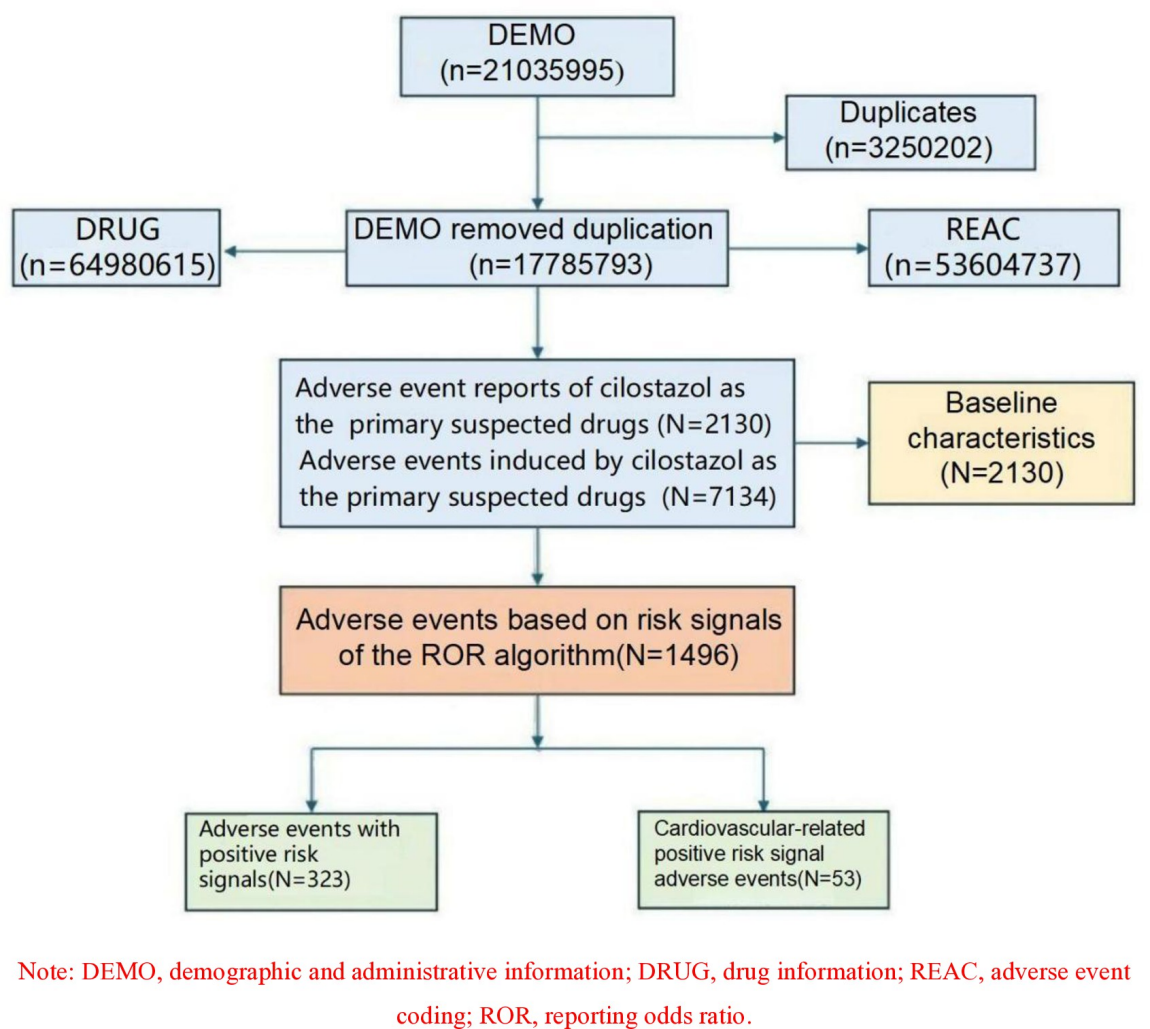
The flowchart of identifying cilostazol related adverse events in FAERS database. Note: DEMO, demographic and administrative information; DRUG, drug information; REAC, adverse event coding; ROR, reporting odds ratio.

Based on the FAERS database, AE reports related to cilostazol were obtained. Following the exclusion of irrelevant and duplicate reports, a total of 2,130 AE reports and 7,134 AEs were identified where cilostazol was the primary suspect drug. Please refer to Fig 1 for a detailed depiction of the process.

#### 3.1.2 Basic information of adverse drug events

**Fig 2:**
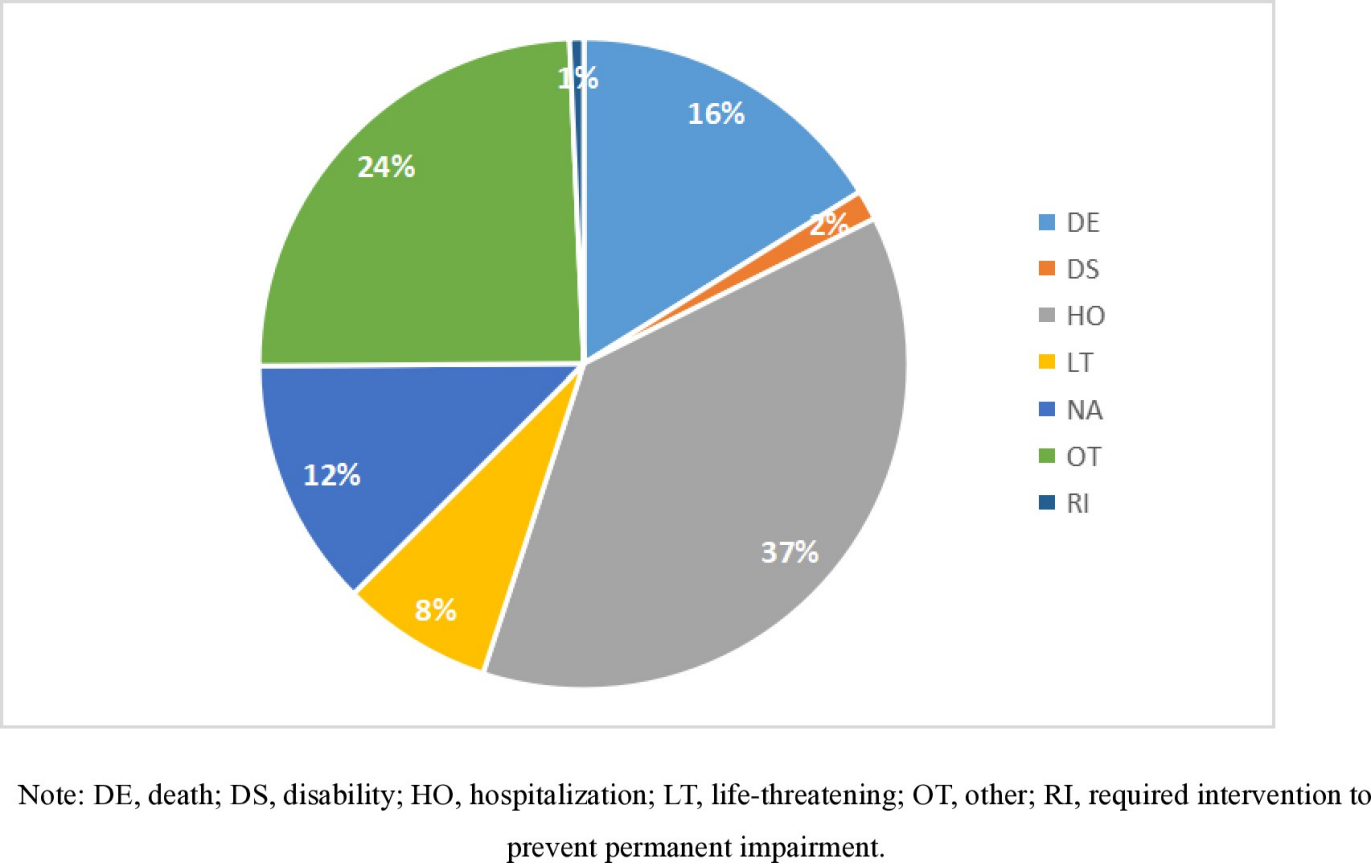
Proportion of adverse drug outcomes. Note: DE, death; DS, disability; HO, hospitalization; LT, life-threatening; OT, other; RI, required intervention to prevent permanent impairment.

As shown in Table 3, a significant proportion of AE reports for cilostazol involved elderly patients (≥60 years), accounting for 65.3% of the total. The male population(55.9%) showed a higher representation compared to females(31.1%). The majority of the reports were submitted by physicians and other healthcare professionals, comprising 52.5% of the total. In this study, the most severe outcome among the multiple options was selected as the final endpoint. The outcome that contributed to hospitalization or prolonged hospital stay accounted for the highest proportion, with a total of 796 cases (37.4%). Additionally, there were 504 cases (23.7%) that posed a life-threatening risk to patients or associated with patient death. Please refer to Fig 2 for a detailed breakdown of these outcomes.

**Table 3:** Clinical characteristics of reports with cilostazol.

X.	N	%
	(Overall = 2130)	
**SEX**		
Female	662	31.1%
Male	1191	55.9%
NA	277	13.0%
**AGE(year)**		
<18	5	0.2%
18~59.9	268	12.6%
60~85	1255	58.9%
>85	137	6.4%
NA	465	21.8%
**REPORTING OFFICER**		
Physician(MD)	703	33.0%
Other health-professional(OT)	282	13.2%
Pharmacist(PH)	134	6.3%
Consumer(CN)	267	12.5%
Other groups	32	1.5%
NA	712	33.4%
**REPORTING COUNTRY**		
Japan(JP)	1057	49.6%
The United States(US)	243	11.4%
Korea(KR)	173	8.0%
Germany(DE)	146	6.9%
Spain(ES)	86	4.0%
Philippines (PH)	67	3.1%
The United Kingdom(UK)	56	2.7%
China (CN)	53	2.5%
**OUTCOMES**		
Hospitalization(HO)	796	37.4%
Disability(DS)	32	1.5%
Life-threatening(LT)	160	7.5%
Death(DE)	344	16.2%
Required intervention to prevent permanent impairment(RI)	15	0.7%
Other(OT)	520	24.4%
NA	263	12.3%
**REPORTING YEAR**	**N**	
2004	105	
2005	91	
2006	79	
2007	120	
2008	114	
2009	242	
2010	138	
2011	69	
2012	345	
2013	67	
2014	75	
2015	81	
2016	186	
2017	168	
2018	134	
2019	28	
2020	18	
2021	24	
2022	24	
2023	14	
2024	8	

**Fig 3:**
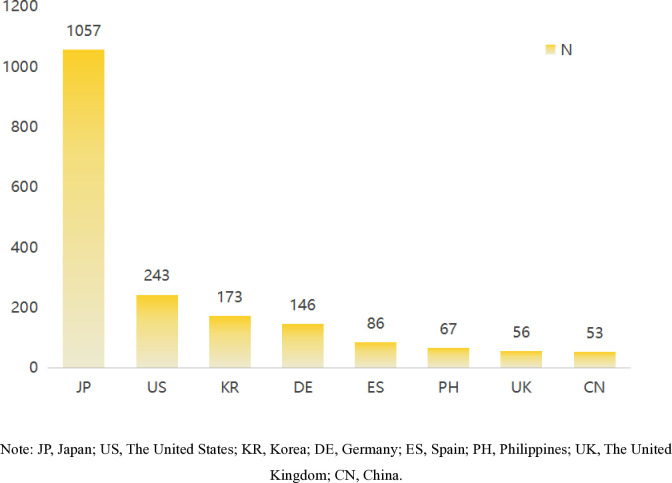
TOP 8 Reporter country. Note: JP, Japan; US, The United States; KR, Korea; DE, Germany; ES, Spain; PH, Philippines; UK, The United Kingdom; CN, China.

The reporting countries were ranked based on the number of reports, with Japan having the highest count, followed by the United States, Korea, Germany, Spain, Philippines, the United Kingdom, and China. For more information, please see Fig 3.

**Fig 4:**
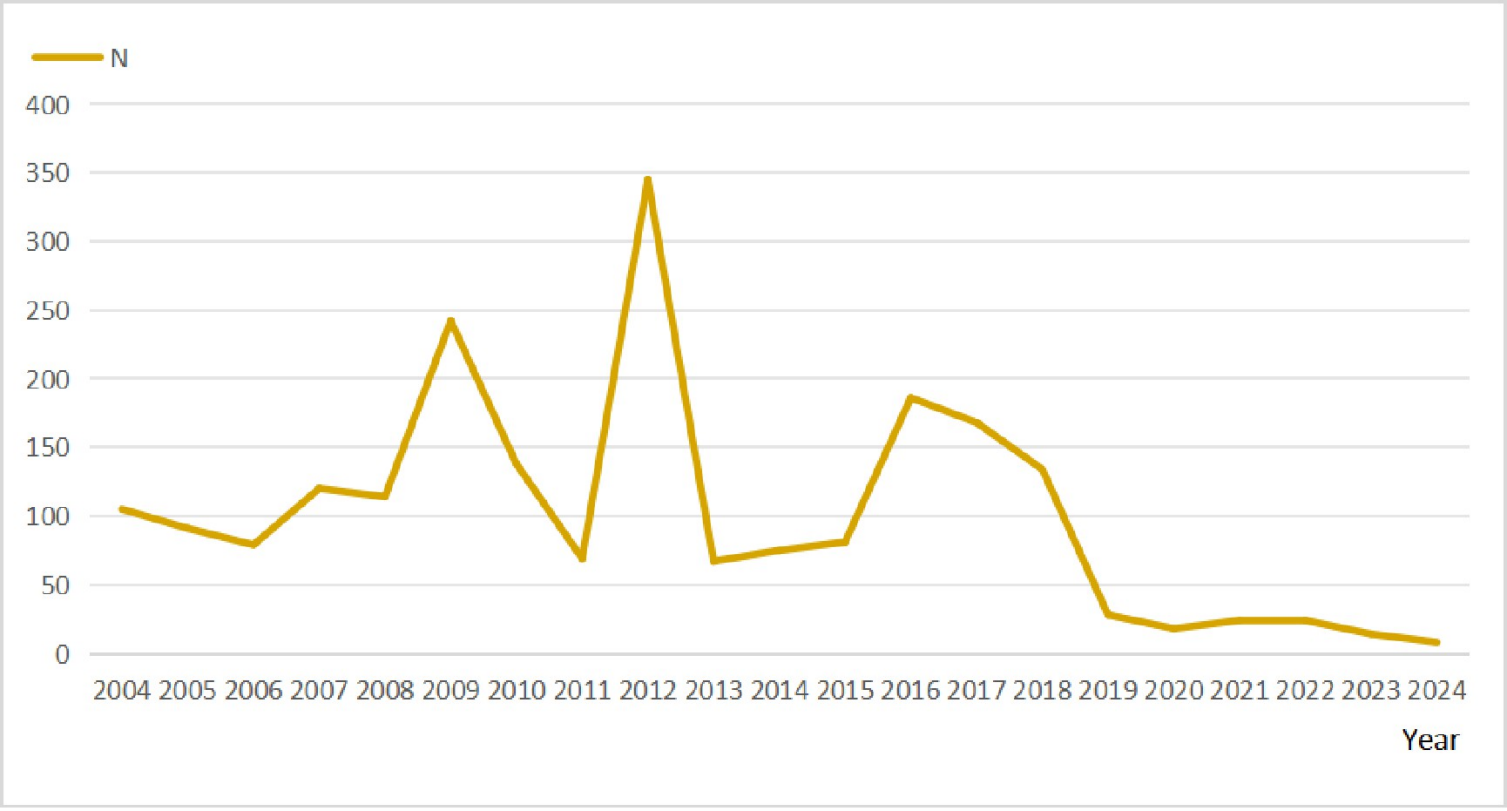
Distribution of adverse events due to cilostazol reported annually.

The annual trend of AE reporting reached its peak in 2012, as illustrated in Fig 4.

### 3.2 Signal strength and reporting frequency of cilostazol

323 positive risk signal AEs screened by cilostazol, involving 23 SOCs. The top 30 PTs were ranked in descending order based on the number of AE reports and the intensity of the risk signals. See Table 4.

**Table 4:** The positive signal strength and the frequency of reports in the top 30 PT of cilostazol.

In the top 30 of PT’s signal strength			In the top 30 of PT’s frequency			
PT	N	ROR(95%Cl)	PT	N	ROR(95%Cl)	%
cardiac vein perforation*	3	3221.2 (832.79–12459.49)	anaemia	99	4.45 (3.65–5.42)	1.39%
basal ganglia haematoma*	3	777.53 (236.8–2553.06)	cardiac failure	82	8.88 (7.14–11.04)	1.15%
cerebral hyperperfusion syndrome*	6	563.95 (245.92–1293.25)	condition aggravated*	79	2.39 (1.91–2.98)	1.11%
arterial restenosis*	5	335.64 (136.98–822.39)	palpitations	67	4.99 (3.92–6.35)	0.94%
putamen haemorrhage	6	175.55 (78.11–394.53)	Fall*	67	1.73 (1.36–2.21)	0.94%
artery dissection*	7	160.01 (75.66–338.4)	cerebral infarction*	66	23.07 (18.1–29.41)	0.93%
vertebral artery stenosis*	3	154.44 (49.23–484.55)	tachycardia	60	5.89 (4.57–7.59)	0.84%
tachycardia paroxysmal	5	146.27 (60.35–354.52)	Pneumonia*	59	1.5 (1.16–1.94)	0.83%
rectal ulcer haemorrhage	4	139.21 (51.76–374.38)	loss of consciousness*	51	3.42 (2.6–4.51)	0.71%
thrombotic cerebral infarction*	6	137.55 (61.32–308.51)	atrial fibrillation	50	4.43 (3.35–5.85)	0.70%
thalamus haemorrhage	12	114.75 (64.86–203.02)	cerebral haemorrhage	46	11.08 (8.29–14.81)	0.64%
haemorrhagic cerebral infarction	9	104.48 (54.09–201.79)	blood pressure decreased	44	5.71 (4.25–7.68)	0.62%
haemobilia	5	104.13 (43.07–251.77)	chest pain	41	1.87 (1.38–2.55)	0.57%
haemorrhagic infarction*	6	89.16 (39.85–199.47)	acute kidney injury*	39	1.72 (1.26–2.36)	0.55%
spinal epidural haematoma*	5	81.54 (33.77–196.9)	gastrointestinal haemorrhage	39	3.83 (2.79–5.24)	0.55%
coronary artery restenosis*	6	76.99 (34.43–172.13)	oedema peripheral	39	2.65 (1.93–3.63)	0.55%
lymphocyte stimulation test positive*	7	64.28 (30.53–135.3)	gastric ulcer haemorrhage	37	54.7 (39.55–75.65)	0.52%
gastroduodenal ulcer*	3	63.52 (20.38–197.93)	myocardial infarction	33	1.52 (1.08–2.14)	0.46%
cerebellar haemorrhage	11	63.28 (34.94–114.6)	interstitial lung disease	32	5.93 (4.19–8.4)	0.45%
brain contusion*	5	57.04 (23.66–137.55)	cardiac failure congestive	32	3.16 (2.23–4.47)	0.45%
gastric ulcer haemorrhage	37	54.7 (39.55–75.65)	acute myocardial infarction	32	8.96 (6.33–12.68)	0.45%
brain stem haemorrhage	6	53.97 (24.17–120.51)	melaena	32	12.25 (8.66–17.35)	0.45%
gastrointestinal erosion*	4	53.31 (19.93–142.58)	haemoglobin decreased	32	2.61 (1.85–3.7)	0.45%
aortic aneurysm rupture*	6	50.69 (22.7–113.18)	platelet count decreased	31	2.48 (1.75–3.54)	0.43%
bleeding time prolonged*	7	47.25 (22.47–99.39)	renal impairment*	29	3.06 (2.12–4.41)	0.41%
helicobacter test positive*	4	45.63 (17.07–121.97)	blood pressure increased	29	1.6 (1.11–2.3)	0.41%
hypochromic anaemia	5	43.16 (17.91–103.98)	alanine aminotransferase increased	29	3.98 (2.76–5.73)	0.41%
gastrointestinal vascular malformation haemorrhagic	3	42.3 (13.6–131.62)	hepatic function abnormal	29	7 (4.86–10.08)	0.41%
lacunar infarction*	12	40.7 (23.07–71.8)	renal failure*	28	1.72 (1.18–2.49)	0.39%
vasogenic cerebral oedema*	3	40.48 (13.01–125.93)	white blood cell count decreased	28	2.21 (1.52–3.2)	0.39%

*Refers to the newly discovered serious adverse reactions.

The number of adverse reactions reported by cilostazol ranked first with anaemia, accounting for 1.39%, ROR (95% Cl) [4.45 (3.65–5.42)]; the AE with the first risk signal intensity was cardiac vein perforation, ROR (95% Cl) [3221.2 (832.79–12459.49)].

In contrast with the existing instructions for cilostazol, the 8 unlisted AEs found based on the number of AE reports were condition aggravated, fall, cerebral infarction, pneumonia, loss of consciousness, acute kidney injury, renal impairment, and renal failure; 19 unlisted AEs based on the risk signal strength, respectively cardiac vein perforation, basal ganglia haematoma, cerebral hyperperfusion syndrome, arterial restenosis, artery dissection, vertebral artery stenosis, thrombotic cerebral infarction, haemorrhagic infarction, spinal epidural haematoma, coronary artery restenosis, lymphocyte stimulation test positive, gastroduodenal ulcer, brain contusion, gastrointestinal erosion, aortic aneurysm rupture, bleeding time prolonged, helicobacter test positive, lacunar infarction, vasogenic cerebral oedema.

### 3.3 Signal of system organ class

The intensity of positive risk signals for cilostazol based on SOC is listed in Table 5. We found that the AEs of cilostazol primarily affected 8 organ systems. The cardiac disorders, ROR (95% Cl) [5.05 (4.7–5.42)], was the first, followed by blood and lymphatic system disorders, vascular disorders, hepatobiliary disorders, investigations, renal and urinary disorders, nervous system disorders, gastrointestinal disorders.

**Table 5:** Signal strength of reports of cilostazol at the SOC level in FAERS database.

SOC	N	ROR	RORL	RORU	ROR(95%Cl)
cardiac disorders	856	5.05	4.7	5.42	5.05 (4.7–5.42)
blood and lymphatic system disorders	277	2.36	2.09	2.66	2.36 (2.09–2.66)
vascular disorders	342	2.3	2.06	2.57	2.3 (2.06–2.57)
hepatobiliary disorders	125	1.94	1.63	2.32	1.94 (1.63–2.32)
investigations	805	1.92	1.79	2.07	1.92 (1.79–2.07)
renal and urinary disorders	220	1.63	1.42	1.86	1.63 (1.42–1.86)
nervous system disorders	844	1.44	1.34	1.55	1.44 (1.34–1.55)
gastrointestinal disorders	733	1.23	1.14	1.33	1.23 (1.14–1.33)

Note: SOC, System Organ Class.

### 3.4 Cardiovascular AEs associated with cilostazol

Out of the 323 positive risk signals identified for cilostazol, a total of 53 AEs were found to be related to the cardiovascular system, accounting for approximately 16.41% of the total signals.

**Fig 5:**
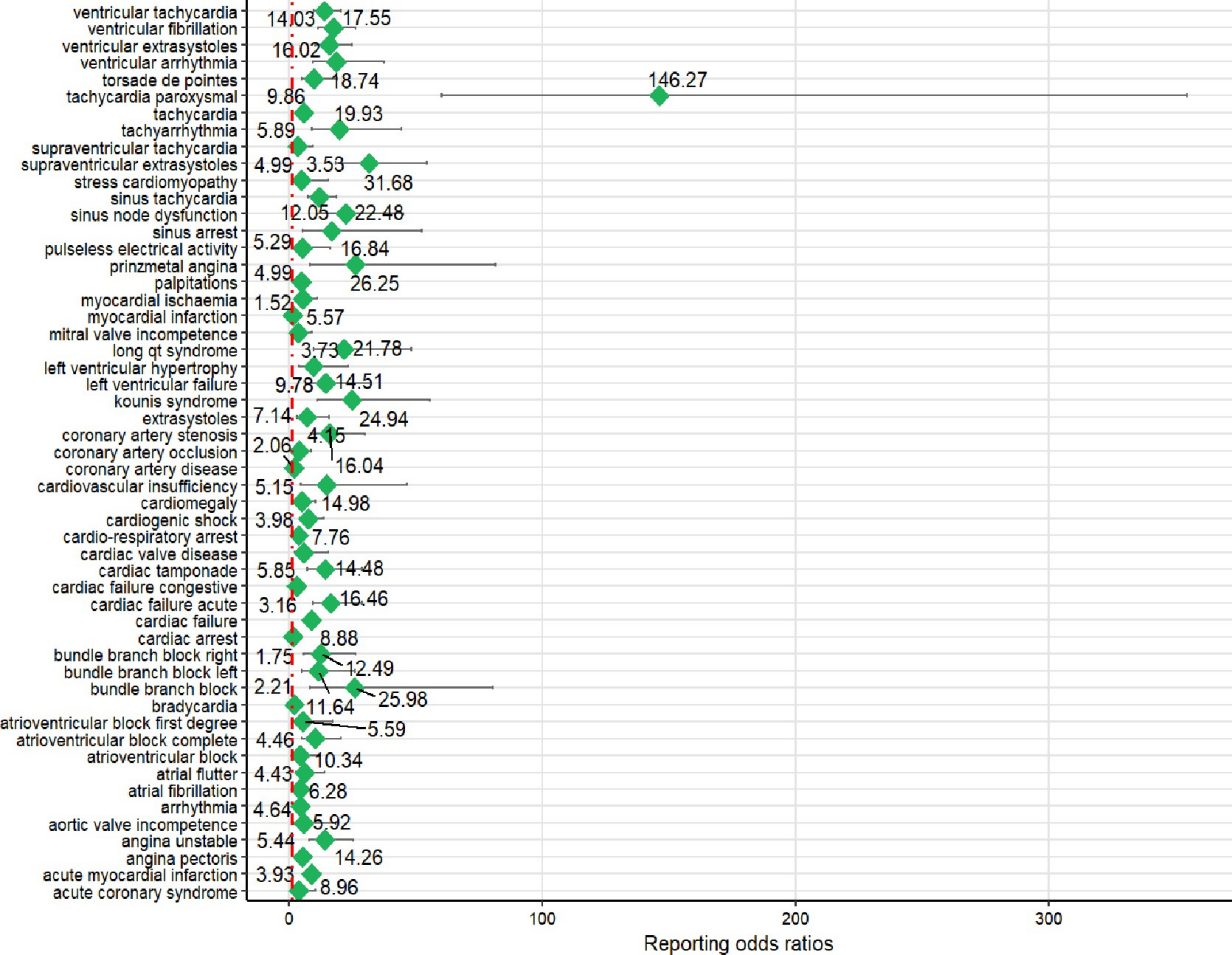
Positive risk signals for cardiovascular adverse events with cilostazol.

Listed below, in descending order of ROR signal strength, are the top 10 cardiovascular AEs: tachycardia paroxysmal, supraventricular extrasystoles, prinzmetal angina, bundle branch block, kounis syndrome, sinus node dysfunction, long qt syndrome, tachyarrhythmia, ventricular arrhythmia, ventricular fibrillation. See Forest Fig 5 for more details.

**Fig 6:**
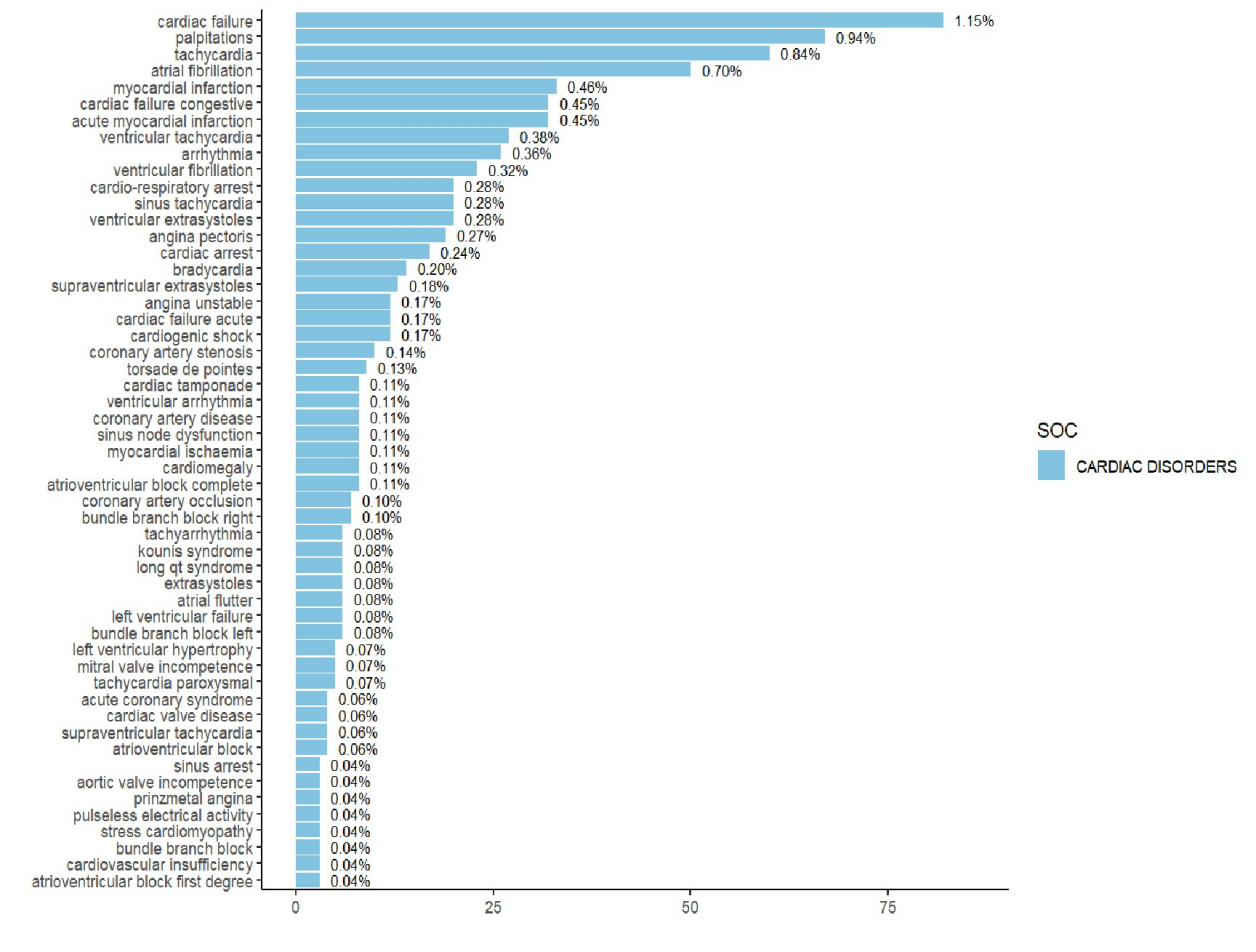
PT distribution of cardiovascular adverse events with cilostazol.

In descending order of the reported numbers, the top 10 were ranked first by cardiac failure, followed by palpitations, tachycardia, atrial fibrillation, myocardial infarction, cardiac failure congestive, acute myocardial infarction, ventricular tachycardia, arrhythmia, ventricular fibrillation. See the PT distribution Fig 6 for more details.

### 3.5 Onset time of events

The onset time and Weighted Signal Proportion (WSP) analysis results for the clinical priority signals of AEs in cilostazol are presented in Table 6. The median time of onset of cilostazol was 62.5 days [interquartile range (IQR): 12–262]. It is worth noting that in the evaluation of WSP analysis, both the shape parameter β and its upper limit of the 95% confidence interval (CI) are less than 1. This observation indicates that the clinical priority signals of cilostazol exhibit an early decline pattern.

**Table 6:** Time-to-onset analysis for signals with cilostazol.

Prioritization				Weibull distribution				Failure type
	Case	TTO(days)		Scale parameter		Shape parameter		
	n	Median (IQR)	Min–max	α	95% CI	β	95% CI	
**Cilostazol**	1090	62.5(12–262)	1–5757	162.30	145.16–179.44	0.61	0.58–0.64	Early failure

Note: n, number of cases with available time-to-onset; IQR, interquartile range; TTO, Time-to-onset.

**Fig 7:**
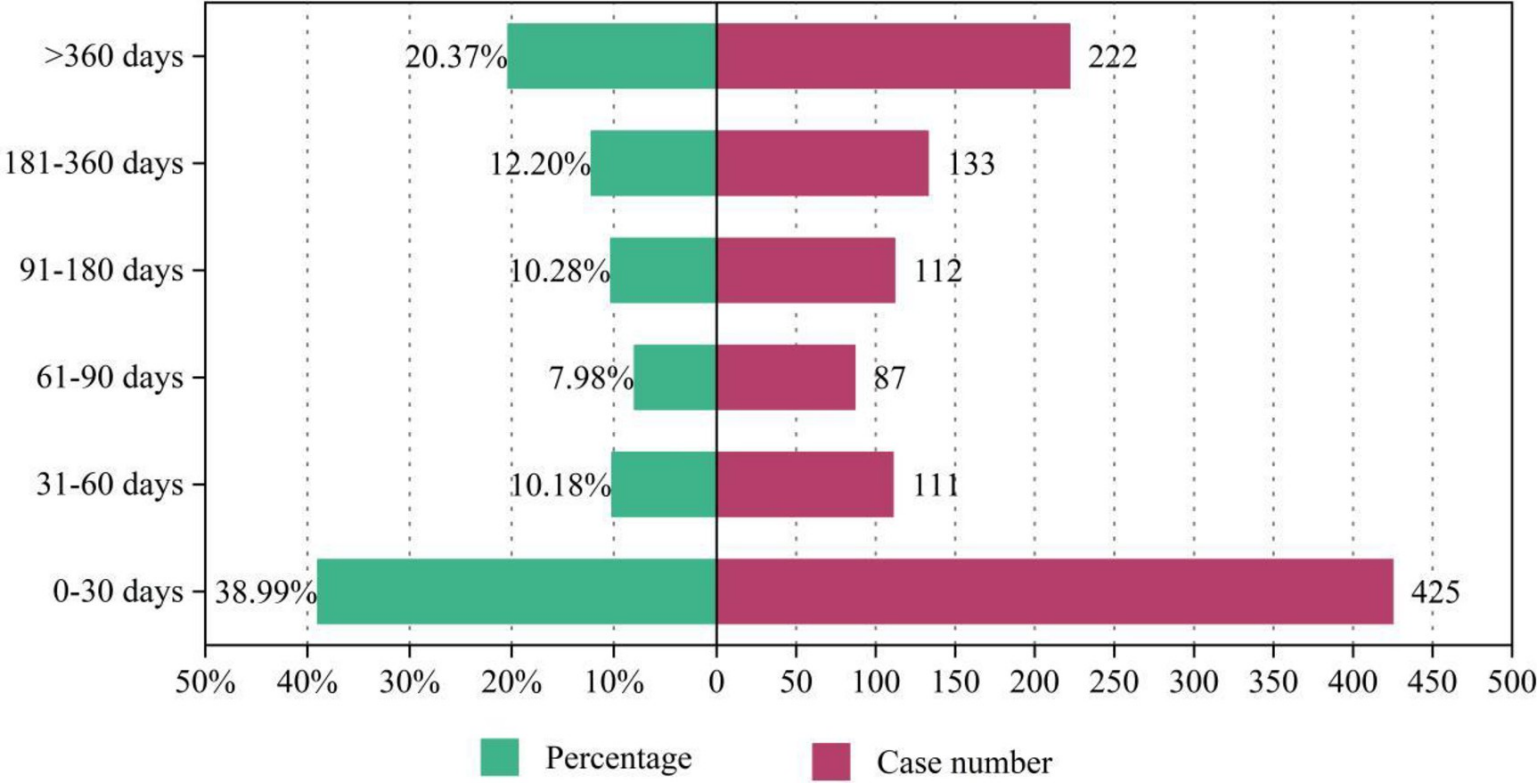
Time-to-onset of cilostazol related adverse events.

By retrieving and summarizing the AE occurrence timeline for cilostazol from the FAERS database, detailed information can be found in Fig 7. The AEs associated with cilostazol predominantly occur within the first month of treatment (N = 425, 38.99%). However, in the long term, beyond the initial month, although the AE rate decreases, there is a gradual increase in the occurrence rate of AEs after the third month of medication. It is important to note that a certain proportion of AEs (n = 222, 20.37%) were reported after long-term treatment with cilostazol (exceeding 360 days).

## 4 Discussion

Cilostazol is a quinolinone derivative that inhibits the activity of phosphodiesterase III, thereby suppressing the degradation of cAMP. This mechanism leads to increased levels of cAMP within blood platelets and vascular endothelium, exerting inhibitory effects on platelet aggregation and vasodilation. It is indicated for the treatment of intermittent claudication and other ischemic symptoms caused by chronic arterial occlusion [21]. The American College of Cardiology and the American Heart Association guidelines approved cilostazol as a first-line treatment for intermittent claudication in 1999 and recommended its use to improve walking distance in patients with peripheral vascular disease [22]. By analyzing data from the FAERS database, we collected and evaluated the post-marketing pharmacovigilance of cilostazol. Our study uncovered AEs associated with cilostazol that were not mentioned in the drug label, enhancing healthcare professionals’ understanding of the drug’s safety profile.

A significant proportion of AE reports for cilostazol involve elderly patients. It is noteworthy that population-based studies have indicated that cilostazol is often prescribed in conjunction with other drugs with potential interactions in the elderly population, particularly those with cardiovascular diseases [23]. The majority of reporters are physicians and other healthcare professionals, which may be attributed to the trust patients place in their doctors and their ability to promptly report drug-related adverse reactions to physicians. Encouraging the active involvement of clinical pharmacists in the entire medication process and strengthening patient medication monitoring can help identify problems and provide appropriate management recommendations, thus further enhancing clinical drug safety. Additionally, this observation indirectly reflects the authenticity and accuracy of the reported AEs. The majority of AE reports originate from Asian countries, with Japan being the most prominent contributor. However, despite the numerous clinical benefits observed, cilostazol’s usage in the United States is relatively low compared to other regions. This discrepancy may be attributed to the fact that most of the evidence supporting cilostazol’s efficacy is derived from Asian populations [24].

The mining results have identified 323 positive risk signals for AEs associated with cilostazol, spanning 23 SOCs. Comparing with the existing cilostazol label, based on the number of AE reports, we have identified 8 adverse reactions that are not currently included. These include: condition aggravated, fall, cerebral infarction, pneumonia, loss of consciousness, acute kidney injury, renal impairment, renal failure. Furthermore, based on the strength of risk signals, we have found 19 AEs that are not currently listed. These are: cardiac vein perforation, basal ganglia haematoma, cerebral hyperperfusion syndrome, arterial restenosis, artery dissection, vertebral artery stenosis, thrombotic cerebral infarction, haemorrhagic infarction, spinal epidural haematoma, coronary artery restenosis, lymphocyte stimulation test positive, gastroduodenal ulcer, brain contusion, gastrointestinal erosion, aortic aneurysm rupture, bleeding time prolonged, helicobacter test positive, lacunar infarction, vasogenic cerebral oedema.

Among the positive risk signals based on the SOC classification, cilostazol has been found to primarily impact 8 organ systems, with cardiac disorders being the most affected. Within the identified positive risk signals for AEs associated with cilostazol, 53 are related to cardiovascular events, representing a significant proportion. When ranked by the number of reported cases, cardiac failure is the most prominent AE, aligning with the black box warning issued by the FDA for cilostazol. These findings underscore the importance of closely monitoring and assessing the safety profile of cilostazol, particularly in relation to cardiac disorders and other cardiovascular events. It is crucial for healthcare professionals and regulatory authorities to consider these previously unreported adverse reactions and the strength of risk signals to ensure the safe and effective use of cilostazol.

### 4.1 Association of AEs with cilostazol

#### 4.1.1 The aspect of the cardiovascular toxicity of cilostazol

Black Box Warning from the FDA [25,26]: Cilostazol is contraindicated in patients with any degree of heart failure. Cilostazol and its numerous metabolites inhibit phosphodiesterase III. Several drugs with this pharmacological action have been associated with reduced survival in patients with congestive heart failure of class III or IV when compared to placebo. Given the previous association between the use of other phosphodiesterase III inhibitors and increased mortality in patients with class III or IV heart failure [27], cilostazol is not recommended for use in patients with any degree of heart failure.

Cilostazol has been shown to increase the heart rate by approximately 5 to 7 beats per minute, which may elevate the risk of cardiac events in certain patients, such as those with stable angina pectoris [28]. Due to the potential for cilostazol to induce angina pectoris through its heart rate-increasing effect, close observation and inquiry regarding anginal symptoms (chest pain) are particularly important [29]. Research findings [25] indicate that the utilization of elevated dosages reveals left ventricular endomyocardial thickening and coronary artery lesions, with safe dosages being 30 mg/kg/day and 12 mg/kg/day respectively. Additionally, certain experimental studies have corroborated that the elevation of intracellular cAMP concentrations induced by drugs exerts direct toxic effects on myocardial cells and enhances the electrophysiological mechanisms underlying arrhythmogenesis [30–32]. In clinical research, the use of cAMP modulators in treatment has been associated with accelerating disease progression and the occurrence of severe ventricular arrhythmias [33,34].

The European Medicines Agency (EMA) [35] released data from nearly 14,000 suspected adverse drug reaction reports (with a background exposure of over 6 million patient-years worldwide) and 4,000 non-interventional study events, confirming the known adverse reaction profile of cilostazol in clinical trials. The most frequently reported cardiovascular events included palpitations (accounting for approximately 5% of total spontaneous reporting cases) and tachycardia (accounting for approximately 5% of total spontaneous reporting cases). In the CASTLE study [36], a post-marketing investigation was conducted to assess the long-term cardiovascular safety of cilostazol. The primary endpoint of the study was all-cause mortality. Among the cilostazol group, there were 49 cases of death, with 12 cases related to cardiovascular disease. In the placebo group, there were 52 cases of death, with 3 cases related to cardiovascular disease. This highlights the potential negative impact on patient survival rates associated with the use of this medication. Physicians should take this into consideration when evaluating treatment options and avoid prescribing cilostazol for patients with heart failure. This approach ensures patient safety and allows for close monitoring of any cardiovascular AEs related to cilostazol.

#### 4.1.2 With the hepatobiliary system, kidney and urinary system

The exact mechanisms underlying drug-induced liver and kidney injury remain unclear. However, the following are potential explanations [37–39]: ① Drug metabolism: Cilostazol undergoes extensive metabolism by hepatic cytochrome P-450 enzymes, primarily CYP3A4, and to a lesser extent, CYP2C19. The main route of excretion is through urine (accounting for 74% of elimination). Individual variations in metabolic capacity or specific genetic variations may exist, leading to abnormal drug metabolism or accumulation, thereby triggering liver and kidney injury; ② Immune reaction: Certain individuals may develop an immune reaction to cilostazol or its metabolites, resulting in liver and kidney injury. Immune reactions can trigger inflammatory responses and tissue damage. It is important to emphasize that these explanations are speculative, and the specific mechanisms of drug-induced liver and kidney injury may vary among individuals. When using cilostazol, physicians should closely monitor the liver and kidney function of patients and remain vigilant for any symptoms or signs associated with liver and kidney injury. If necessary, dose adjustments or alternative treatment options may be considered to ensure patient safety.

#### 4.1.3 With the blood and lymphatic system, blood physical and chemical indicators

Cilostazol exerts its effects by blocking the activity of phosphodiesterase III, thereby inhibiting the degradation of intracellular cAMP in platelets. This leads to an elevation in cAMP levels within platelets. Increased cAMP levels inhibit platelet activation and aggregation, consequently reducing the risk of platelet adhesion and thrombus formation. However, this antiplatelet action may also lead to a decreased ability to form blood clots, thereby increasing the risk of bleeding [40].

#### 4.1.4 With the neurological aspects

Central nervous system adverse reactions are relatively uncommon in patients using cilostazol. This may be attributed to the potential impact of cilostazol’s antiplatelet action on hemorheology, including increasing blood flow velocity and reducing blood viscosity [40]. These changes may have certain effects on cerebral blood supply, potentially leading to symptoms such as headache, dizziness, or other flow-related manifestations. Cilostazol’s effects on the cardiovascular system, such as increased blood pressure, are noteworthy. In certain cases, it may result in elevated blood pressure or blood pressure fluctuations, which could have adverse effects on cerebral blood vessels. Hypertension is a recognized risk factor for cerebral vascular hemorrhage. In individuals who already have other risk factors for bleeding, the occurrence of bleeding in cerebral vessels can induce a range of neurological changes [41].

#### 4.1.5 With the gastrointestinal system

Gastrointestinal adverse reactions are relatively common in patients using cilostazol, but they are typically mild and transient. Cilostazol may have a stimulating effect on the gastric mucosa, leading to symptoms such as gastric discomfort, nausea, and vomiting. This may be related to cilostazol’s chemical properties, direct contact with the gastric mucosa, or its influence on gastric acid secretion [42]. Cilostazol may achieve its antiplatelet effects by inhibiting gastric acid secretion. However, the reduction in gastric acid may result in bacterial overgrowth in the gastrointestinal tract, leading to symptoms of gastrointestinal discomfort, bloating, and diarrhea [43]. The antiplatelet action of cilostazol may also have an impact on gastrointestinal blood flow, especially when used at high doses or for prolonged periods [40]. This may cause mucosal ischemia in the gastrointestinal tract, resulting in gastrointestinal AEs such as gastritis and ulcers.

### 4.2 Study significance and limitations

This study is based on the FAERS database to explore and analyze AE signals associated with cilostazol. It investigates the risks of AEs related to cilostazol, particularly cardiac toxicity, and other meaningful AEs, with the aim of providing some reference for improving the safety of clinical drug use. The FAERS database is a spontaneous reporting system. Due to its inherent limitations, there may be underreporting, duplicate reports, and incomplete case information. The lack of information on underlying diseases and concomitant medications can potentially impact the results. Additionally, media attention and recent publications on drug AEs may influence reporting behavior [44]. Furthermore, disproportionality analysis provides a statistical assessment of signal strength but does not establish actual risk. The causality between the drug and AEs does not require confirmation in the FAERS database, and some AE reports may have incomplete clinical and medical information, such as gender and age, as observed in this study. Therefore, establishing such causal relationships would require further validation [45]. Despite the limitations of the FAERS database in pharmacovigilance research, it provides a comprehensive characterization of AE signals associated with cilostazol and uncovers some unexpected AE signals, which may serve as a basis for further clinical research on cilostazol.

## 5 Conclusion

In this study, a pharmacovigilance analysis of cilostazol was conducted using the FAERS database. Disproportionality analysis was employed to detect the ROR signal associated with AEs, and the number of reported AEs was quantified to supplement those not mentioned in the product labeling. The mining results revealed that cilostazol screened out 323 positive risk signal AEs involving 23 SOCs. Comparing with the existing cilostazol label, based on the number of adverse reaction reports, 8 AEs that are not currently included were identified, and based on the strength of risk signals, 19 AEs that are not currently listed were discovered. Cilostazol’s positive risk signal discoveries based on the SOC mainly affected 8 organ systems. Additionally, a statistical analysis was performed to assess the risk of cardiac toxicity associated with cilostazol, confirming that any degree of heart failure is included as a black-box warning entry issued by the FDA. Consequently, close attention should be paid to the long-term safety and cardiovascular toxicity of cilostazol, providing evidential references for the further rational and safe clinical use of this medication.

## Supporting information

S1 Table Positive risk signal adverse events screened by cilostazol. (XLSX)

## Abbreviations

IC: Intermittent claudication
PAD: peripheral arterial disease
PDE3: phosphodiesterase 3
cAMP: cyclic adenosine monophosphate
SMCs: smooth muscle cells
FDA: U.S. Food and Drug Administration
FAERS: FDA Adverse Event Reporting System
SRS: spontaneous reporting system
DEMO: demographic and administrative information
DRUG: drug information
REAC: coded for the adverse events
OUTC: patient outcomes
RPSR: report sources
THER: therapy start dates and end dates for reported drugs
INDI: indications for drug administration
AE: adverse event
MedDRA: Medical Dictionary for Regulatory Activities
SOC: system organ class
PT: preferred term
DPA: disproportionality analysis
ROR: reporting odds ratio
PRR: proportional reporting ratio
CI: confidence interval
WSP: Weighted Signal Proportion
IQR: interquartile range
EMA: European Medicines Agency
